# Influence of Rapid Urbanization on Thyroid Autoimmune Disease in China

**DOI:** 10.1155/2021/9967712

**Published:** 2021-06-02

**Authors:** Yingchao Chen, Bing Han, Jie Yu, Yi Chen, Jing Cheng, Chunfang Zhu, Fangzhen Xia, Ningjian Wang, Yingli Lu

**Affiliations:** Institute and Department of Endocrinology and Metabolism, Shanghai Ninth People's Hospital Affiliated to Shanghai Jiao Tong University School of Medicine, Shanghai, China

## Abstract

**Background:**

The prevalence of autoimmune thyroid diseases (AITDs), especially Hashimoto's thyroiditis (HT), has increased dramatically in China. Moreover, China is experiencing the largest scale of urbanization in the world. We intended to explore the relationship between rapid urbanization and HT.

**Methods:**

A total of 2946 subjects in Zhejiang Shangyu (SY) (*n* = 1546) and Jiangsu Nanjing (NJ) (*n* = 1400) were enrolled in this study. Serum TPOAb, TGAb, and thyrotropin (TSH) were measured, and ultrasonography of the thyroid was performed in all subjects. DNA was extracted from all subjects, and four SNPs were selected for genotyping. Generalized multifactor dimensionality reduction (GMDR) was used to screen the best interaction between genetic factors and environment factors.

**Results:**

TPOAb and TGAb concentrations were higher in NJ than in SY (34.60 vs. 14.00 IU/ml and 21.05 vs. 7.50 IU/ml). People in NJ also had higher TPOAb and TGAb positivity rates than those in SY (7.8% vs. 12.7% and 8.7% vs. 16.3%). Logistic regression analysis indicated that rapid urbanization was an independent risk factor for TPOAb (OR = 1.473) and TGAb (OR = 1.689). Genotype TT in rs11675434 was associated with an increased risk of TPOAb positivity both in SY (OR = 2.955) and in NJ (OR = 1.819). GMDR analysis showed a two-locus model (SNP2 × urbanization) and a three-locus model (SNP2 × SNP3 × urbanization), which had testing accuracies of 56.88% and 57.25%, respectively (*P* values were 0.001 and 0.001).

**Conclusion:**

Rapid urbanization influences the incidence of TPOAb and TGAb positivity. We should pay more attention to thyroid autoimmune disease in areas of China experiencing rapid urbanization.

## 1. Introduction

The prevalence of thyroid diseases is experiencing a rapid increase [1, 2]. In Scotland, the prevalence of all thyroid diseases increased from 2.3% in 1994 to 3.8% in 2001, representing a 63% increase [3]. Autoimmune thyroid diseases (AITDs), including Hashimoto's thyroiditis (HT) and Graves' disease (GD), are frequently observed autoimmune disorders, affecting 2–5% of the general population [4]. In urban areas of China, the prevalence of AITDs is 10.5% in men and 21.4% in middle-aged women [5]. HT is considered the most common autoimmune disease [6]. High levels of thyroid autoantibodies against thyroid peroxidase (TPOAb) are present in 90% of patients with HT and serve as a clinical marker for the early detection of HT [7]. A survey conducted in Italy showed that the prevalence of HT was higher in 2010 than in 1995, increasing markedly from 3.5% to 14.5% [2]. The findings of a study conducted in Denmark indicated significant increases in the prevalence of TPOAb positivity from 14.3% to 23.8% [8]. Similarly, the prevalence of TPOAb positivity increased from 9.09% in 1999 to 12.6% in 2011 in China [1].

China is urbanizing at an extremely high speed [9]. Urbanization, especially rapid urbanization, appears to be a double-edged sword in terms of the effect on people's daily life [10]. Although the process can bring great opportunity for technological development [9], urbanization has some adverse consequences, such as air pollution, sedentary lifestyles, and increased life stress, thus affecting the residents' health status [10]. In our previous study, the prevalence of diabetes, hyperlipidemia, and nonalcoholic fatty liver disease (NAFLD) increased in the area of rapid urbanization [11]. Increasing trends in cardiovascular mortality were also reported among indigenous populations during a rapid urbanization process [12]. As people's life patterns changed, urbanization was found to be associated with an increasing incidence of breast cancer in Chinese women [13]. Regarding mental aspects, urbanization might promote positivity and happiness by stimulating the protective effect of neighborhood-level reciprocity and social group membership, but it can also result in mental illnesses by reducing neighborhood social capital [14].

It is widely accepted that the complex interactions of genetic susceptibility, environmental factors, and immune disorders contribute to the development of HT [15]. There are considerable studies regarding the relationship between HT and genetic predisposition. However, there have been few studies concerning rapid urbanization and thyroid autoimmune disease.

Hence, in this study, we intended to confirm that urbanization may influence the prevalence of HT and aimed to clarify whether there are interactions between SNPs and urbanization. This exploration will enable us to better understand the mechanism of HT and the influence of rapid urbanization on HT.

## 2. Subjects and Methods

### 2.1. Study Population

**Figure 1:**
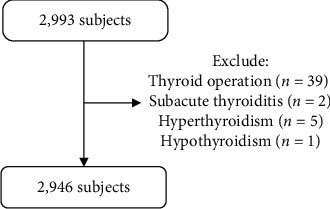
Flowchart of this study.

This is a substudy of SPECT-China (registered number: ChiCTR-ECS-14005052, http://www.chictr.org), representing a population-based cross-sectional survey regarding the prevalence of metabolic diseases and risk factors in eastern China. A stratified cluster sampling method was applied. In this study, 1,569 subjects in Zhejiang Shangyu (SY) and 1,427 subjects in Jiangsu Nanjing (NJ) were recruited. Adults who had lived in their current residence for 6 or more months were selected and enrolled in our study. We performed our investigation together with village and community administrators of residence registration. Information of participants in this investigation was collected by house-to-house visit. Urbanization of SY was gradually achieved by urban expansion and migration from rural to urban areas. However, urbanization in NJ was achieved by in situ urbanization. Therefore, in the past 10 years, NJ experienced rapid urbanization, whereas SY developed relatively slowly. The exclusion criteria included the following: thyroidectomy (*n* = 39) hyperthyroidism (*n* = 5), subacute thyroiditis (*n* = 2), or hypothyroidism (*n* = 1). Finally, the current study was based on a total of 2,946 subjects (1,546 in SY and 1,400 in NJ) (Figure 1). The study protocol was approved by the Ethics Committee of Shanghai Ninth People's Hospital. Written informed consent was obtained from all participants.

### 2.2. Genomic DNA Extraction and Genotyping

Seven SNPs were selected according to previous literature [4], including rs10944479, rs11675434, rs1230666, rs3094228, rs653178, rs9277555, and rs301799. DNA was extracted from peripheral white blood cells with a DNA Blood Mini Kit (DP603; Tiangen Biotech Co., Ltd., Beijing, China) using an automated nucleic acid extraction instrument (YOSE-S32; Tiangen Biotech Co., Ltd.). Specific assays were designed with the Geneious Pro (version 4.8.3; https://www.geneious.com/). Mass determination was carried out with the JUNO, and data acquisition was performed by Fluidigm SNP Genotyping Analysis version 4.1.3 software (Fluidigm Corporation, South San Francisco, CA). The call rates of all SNPs were 98%. Rs10944479, rs653178, and rs1230666 had only one allele; therefore, these three SNPs were excluded. Genotype distribution of 4 SNPs was in the Hardy–Weinberg equilibrium. The nucleotide sequences of primers and descriptions of the 4 SNPs are shown in Supplemental Table 1.

### 2.3. Assessment of Indexes

Blood sample collection, storage, and shipment have been described previously [11]. Serum TPOAb, TGAb, and thyrotropin (TSH) were measured by chemiluminescence immunoassays (Immulite 2000; Siemens, Munich, Germany). Demographic information and lifestyle risk factors were collected by trained staff using standard questionnaires. Body mass index (BMI) was calculated as weight (kg)/height squared (m^2^). TPOAb or TGAb higher than 60 IU/ml was defined as serum TPOAb or TGAb positivity. Diabetes was defined as self-reported diabetic history or HbA1c 6.5% or greater.

### 2.4. Statistical Analysis

Statistical analysis was performed using IBM SPSS Statistics, Version 22 (IBM Corporation, Armonk, New York). Continuous variables are expressed as mean ± SD or median with the interquartile range (IQR). Categorical variables are expressed as a percentage (%).The Mann–Whitney *U* test and independent sample *t*-test were used for continuous data with skewed distribution and normal distribution between SY and NJ. The Pearson chi-square test was used for categorical variables. We used binary regression analyses to investigate the association between TPOAb/TGAb positivity and urbanization level. Model 1 was adjusted for age and sex. Model 2 was further adjusted for smoking. Model 3 was further adjusted for BMI and diabetes. Generalized multifactor dimensionality reduction (GMDR) (http://sourceforge.net/projects/gmdr/) was used to screen the best interaction combination among the 4 SNPs and urbanization. In GMDR analysis, 633 subjects were excluded because they lack both of the four SNPs data. All reported *P* values were two-tailed, and those less than 0.05 were considered statistically significant.

## 3. Results

### 3.1. Comparison between Different Locations

In this study, 1,546 subjects, including 694 males (age 54.18 ± 13.38 years) and 852 females (age 53.44 ± 13.60 years), in SY and 1,400 subjects, including 446 males (age 56.88 ± 11.86 years) and 954 females (age 55.36 ± 12.45 years), in NJ were recruited. There was a significant difference in BMI between individuals in SY and those in NJ (23.03 ± 3.23 vs. 25.52 ± 3.29 kg/m^2^, *P* < 0.001).

### 3.2. Comparison of TSH, TPOAb, and TGAb in the Two Locations

As shown in Table 1, TPOAb and TGAb concentrations were higher in NJ than in SY (34.60 vs. 14.00 IU/ml and 21.05 vs. 7.50 IU/ml). Residents in NJ had higher TSH levels than those in SY (3.13 ± 5.55 vs. 2.38 ± 1.46). This trend was also observed in both sexes. As shown in Supplemental Table 2, people in NJ had higher TPOAb and TGAb positivity than those in SY (7.8% vs. 12.7% and 8.7% vs. 16.3%). However, females, but not males, in NJ had higher TPOAb and TGAb positivity than those in SY (*P* = 0.003 and *P* < 0.001).

### 3.3. Logistic Regression Analysis of Risk Factors for TPOAb and TGAb Positivity

TPOAb positivity was used as a dependent factor, and other factors were selected as independent factors. The incidence of TPOAb positivity in NJ was 56.4% higher than that in SY after adjusting for sex and age. After adjusting further for smoking, TPOAb positivity in NJ was still 58.4% higher than that in SY. After further adjustment for BMI and diabetes, the incidence of TPOAb positivity was 47.3% higher in NJ than in SY. In addition, in all three models, sex was the most significant risk factor for TPOAb positivity (Table 2). When TGAb positivity was used as a dependent factor, the incidence of TGAb positivity in NJ was 80.5% higher than that in SY in model 1, 82.6% higher in model 2, and 68.9% higher in model 3 (Table 3).

### 3.4. Relationship of 4 SNPs with TPOAb Positivity

Logistic regression analysis indicated that genotype TT of rs11675434 was associated with an increased risk of TPOAb positivity both in SY (OR = 2.955) and NJ (OR = 1.819). In SY, genotype AG of rs9277555 was associated with an increased risk of TPOAb positivity (OR = 1.912). Genotype CC of rs301799 was associated with an increased risk of TPOAb positivity in NJ (OR = 2.485). There was no significant relationship between rs3094228 and TPOAb positivity in both SY and NJ (Table 4).

### 3.5. GMDR Analysis of Gene-Gene and Gene-Urbanization Relationships

The cross-validation consistency and the testing accuracy for each of the models were determined by GMDR analysis. The two-locus model (SNP2 × urbanization) and three-locus model (SNP2 × SNP3 × urbanization) had testing accuracies of 56.88% and 57.25%, respectively (*P* values were 0.001 and 0.001). However, there were no significant differences between different SNPs. This study showed evidence of a gene-urbanization interaction effect. The analysis indicated that rapid urbanization influenced TPOAb positivity depending on the genotypes at SNP2 and SNP3 (Table 5).

## 4. Discussion

In this study, we compared the incidence of TPOAb and TGAb positivity in two cities with different rapid urbanization. We found that rapid urbanization was an independent risk factor for both TPOAb and TGAb positivity. Furthermore, rapid urbanization could interact with susceptibility genes and promote an increase in TPOAb positivity.

HT is a common organ-specific autoimmune disorder characterized by the infiltration of the thyroid gland by inflammatory cells. HT causes destruction of thyroid follicles, which leads to hypothyroidism [16]. A diagnosis of HT is dependent on both ultrasonography and the assessment of autoantibodies (TPOAb or TGAb) [17]. Typically, HT is considered to arise from interactions between genetic factors and environmental factors [18]. Moreover, the incidence of thyroid autoimmune disease is increasing in recent years in China. According to a recent study, iodine deficiency has been successfully eliminated, but other thyroid disorders caused by increased iodine intake should be considered in China [1]. However, there have been few studies concerning rapid urbanization and thyroid disease.

Previously, many studies have reported a genetic predisposition to autoimmune thyroiditis. Medici et al. performed GWAS meta-analyses for TPOAb positivity and for TPOAb serum levels. Significant associations (*P* < 5 ×  10^−8^) were detected at TPO (rs11675434), ATXN2 (rs653178), and BACH2 (rs10944479) for TPOAb positivity and at TPO (rs11675434), MAGI3 (rs1230666), and KALRN (rs2010099) for TPOAb levels [4]. Schultheiss et al. studied participants with European ancestry in 3 independent prospective population-based studies. In the beginning, we sequenced 7 SNPs. But three of them (rs10944479, rs653178, and rs1230666) had only one allele. Therefore, they were excluded in the study. Four novel genetic loci (rs301799 near *RERE*, rs3094228, rs1894407, and rs9277555 in the *HLA* region) were identified as being associated with TPOAb concentrations [19]. However, in our study, genotype TT in rs11675434 was associated with an increased risk of TPOAb positivity both in SY (OR = 2.955) and in NJ (OR = 1.819). Genotype AG of rs9277555 was associated with an increased risk of TPOAb positivity (OR = 1.912) only in SY. However, in overall population, genotype AG showed no significant association with TPOAb positivity (OR = 1.299, 95% CI 0.933, 1.808). Genotype CC of rs301799 was associated with an increased risk of TPOAb positivity both in NJ and overall population (OR = 2.209, 95% CI 1.203, 4.058). However, genotype CC showed no significant association with TPOAb positivity in SY, which might be caused by the relatively small sample in TPOAb positive group.

In addition to genetic predisposition, environmental factors, such as nutrients, pollutants, drugs, infections, stress, lifestyle, and socioeconomics, might play an important role in modifying gene expression by epigenetic mechanisms, thus triggering autoimmunity [20, 21]. NOD.H2h4 mice are a spontaneous model of autoimmune thyroiditis. When these mice were housed in conventional conditions, they developed earlier and more severe thyroiditis than those housed under SPF conditions [22].

China is now experiencing the largest scale of accelerated urbanization in the world [23]. Urbanization will cause pollution and stress and lifestyle changes among residents [10]. Researchers found that the prevalence of HT and thyroid antibodies in residents near the petrochemical complex was higher than that in residents in a control area [24]. However, there has been disagreement with this opinion [25]. Furthermore, other pollutants, such as benzene and carbon monoxide organochlorines [26], have been associated with thyroid function. Stress may contribute to the onset of thyroid autoimmunity (TIA), and there is a close link between stress and Graves' disease [27]. However, few reports have studied the relationship between stress and HT. Therefore, the effect of stress on HT might be negligible [27]. Stress may influence the neuroendocrine-immune system, which leads to changes in the internal milieu that regulate homeostasis. Regarding lifestyle, there was a negative association between smoking and the presence of thyroid antibodies [28]. Additionally, discontinuation of smoking is related to an increased risk of TPOAb occurrence [29]. Obesity might also trigger thyroid autoimmunity by dysregulating the pituitary-hypothalamic axis and adipose tissue via leptin [20]. In this study, we used rapid urbanization as the combination of other indicators (such as pollution, socioeconomic status, and chronic stress) rather than discussing them separately.

There are several limitations to our study. First, some residents in SY and NJ did not have genotype data. Second, we did not have data regarding the prevalence of TPOAb and TGAb positivity at the beginning of urbanization. So, methodological criteria of the retrospective data were not available. Third, the samples were collected at different times. Finally, we did not consider urine iodine levels of the residents.

## 5. Conclusion

Our study provides a putative result that rapid urbanization influences TPOAb and TGAb positivity. It suggested that rapid urbanization could interact with SNPs located in TPOAb susceptibility genes and promote an increase in TPOAb positivity. Therefore, thyroid autoimmune disease should be emphasized in rapid urbanization areas in China.

## Figures and Tables

**Table 1:** Comparison of TSH, TPOAb, and TGAb concentrations in two locations.

	TPOAb			TGAb			TSH		
	SY	NJ	*P*	SY	NJ	*P*	SY	NJ	*P*
Overall	14.00 (14.00–14.00)	34.60 (28.00–44.58)	<0.001	7.50 (7.50–15.00)	21.05 (15.00–29.62)	<0.001	2.38 ± 1.46	3.13 ± 5.55	<0.001

*Age*									
<40	14.00 (14.00–14.00)	38.60 (30.30–47.00)	<0.001	7.50 (7.50–7.50)	22.50 (16.43–35.13)	<0.001	2.15 ± 1.14	2.55 ± 1.56	0.004
40–50	14.00 (14.00–14.00)	36.20 (29.00–43.15)	<0.001	7.50 (7.50–16.25)	21.90 (16.98–31.60)	<0.001	2.42 ± 1.61	3.55 ± 10.67	0.053
50–60	14.00 (14.00–14.00)	35.35 (28.00–45.45)	<0.001	7.50 (7.50–7.50)	20.90 (15.00–27.65)	<0.001	2.39 ± 1.36	3.11 ± 5.21	0.009
60–70	14.00 (14.00–14.00)	31.40 (7.75–43.33)	<0.001	7.50 (7.50–7.50)	20.30 (15.00–29.67)	<0.001	2.56 ± 1.70	3.10 ± 2.39	<0.001
≥70	14.00 (14.00–14.00)	28.30 (7.64–41.00)	<0.001	7.50 (7.50–16.00)	19.50 (15.00–28.10)	<0.001	2.12 ± 1.01	3.18 ± 2.30	<0.001

*Gender*									
Male	14.00 (14.00–14.00)	33.45 (28.00–43.80)	<0.001	7.50 (7.50–7.50)	20.05 (15.00–25.11)	<0.001	2.15 ± 1.29	2.43 ± 1.88	0.003
Female	14.00 (14.00–14.00)	34.85 (28.00–45.00)	<0.001	7.50 (7.50–17.00)	22.15 (15.00–39.34)	<0.001	2.56 ± 1.57	3.46 ± 6.58	<0.001

**Table 2:** Logistic regression analysis for risk factors of TPOAb positivity.

	Model 1	Model 2	Model 3
Rapid urbanization	1.564 (1.221, 2.004)	1.584 (1.233, 2.035)	1.473 (1.128, 1.923)
Sex	2.503 (1.865, 3.358)	2.432 (1.666, 3.548)	2.492 (1.699, 3.655)
Age	0.998 (0.989, 1.008)	0.997 (0.987, 1.007)	0.997 (0.987, 1.007)
Smoke		1.032 (0.653, 1.632)	0.999 (0.630, 1.585)
BMI			1.030 (0.992, 1.069)
Diabetes			0.857 (0.586, 1.255)

**Table 3:** Logistic regression analysis for risk factors for TGAb positivity.

	Model 1	Model 2	Model 3
Rapid urbanization	1.805 (1.432, 2.275)	1.826 (1.445, 2.306)	1.689 (1.319, 2.164)
Sex	3.951 (2.917, 5.352)	4.005 (2.783, 5.763)	3.980 (2.760, 5.738)
Age	0.997 (0.988, 1.005)	0.995 (0.987, 1.004)	0.994 (0.985, 1.004)
Smoke		0.908 (0.606, 1.360)	0.907 (0.603, 1.363)
BMI			1.032 (0.996, 1.068)
Diabetes			0.801 (0.559, 1.147)

**Table 4:** Genetic risk estimation for 4 SNPs and TPOAb.

SNP	Genotypes	SY frequencies *N* (%)		OR (95%)	NJ frequencies *N* (%)		OR (95%)
		TPOAb‒	TPOAb+		TPOAb‒	TPOAb+	
SNP1 (rs9277555)	AA	236 (27.5)	14 (17.9)	1 (ref)	297 (25.9)	41 (24.3)	1 (ref)
	AG	432 (50.4)	49 (62.8)	1.912 (1.034, 3.536)	568 (49.6)	85 (50.3)	1.084 (0.728, 1.614)
	GG	189 (22.1)	15 (19.2)	1.338 (0.630, 2.841)	280 (24.5)	43 (25.4)	1.112 (0.704, 1.759)
SNP2 (rs11675434)	CC	427 (48.6)	34 (42.5)	1 (ref)	547 (47.9)	68 (40.2)	1 (ref)
	CT	384 (43.7)	30 (37.5)	0.981 (0.589, 1.634)	481 (42.1)	75 (44.4)	1.254 (0.884, 1.780)
	TT	68 (7.7)	16 (20.0)	2.955 (1.547, 5.643)	115 (10.1)	26 (15.4)	1.819 (1.109, 2.982)
SNP3 (rs301799)	TT	585 (66.3)	49 (62.8)	1 (ref)	820 (71.4)	110 (65.1)	1 (ref)
	CT	278 (31.5)	27 (34.6)	1.160 (0.710, 1.895)	292 (25.4)	47 (27.8)	1.200 (0.832, 1.731)
	CC	20 (2.3)	2 (2.6)	1.194 (0.271, 5.258)	36 (3.1)	12 (7.1)	2.485 (1.255, 4.919)
SNP4 (rs3094228)	AA	600 (69.6)	50 (66.7)	1 (ref)	832 (72.8)	129 (76.3)	1 (ref)
	AG	244 (28.3)	24 (32.0)	1.180 (0.710, 1.964)	282 (24.7)	37 (21.9)	0.846 (0.573, 1.249)
	GG	18 (2.1)	1 (1.3)	0.667 (0.087, 5.098)	29 (2.5)	3 (1.8)	0.667 (0.200, 2.222)

**Table 5:** GMDR analysis for the best interaction combination models.

Locus no.	Best combination	Cross-validation consistency	Testing accuracy	*P* values
Gene-gene interactions				
1	SNP2	10/10	0.5264	0.0547
2	SNP2 × SNP3	10/10	0.5314	0.0547
3	SNP1 × SNP2 × SNP3	10/10	0.5466	0.0547

Gene-urbanization interactions				
1	Urbanization	10/10	0.5610	0.0547
2	SNP2 × urbanization	10/10	0.5688	0.001
3	SNP2 × SNP3 × urbanization	10/10	0.5725	0.001

## Data Availability

The data used to support the findings of this study are available from the corresponding author upon reasonable request.

## Supplementary Materials

Supplementary Materials Supplementary Table 1: description of four SNPs. Supplementary Table 2: comparison of TPOAb and TGAb positivity in two locations.

## References

[1] Shan Z., Chen L., Lian X. (2016). Iodine status and prevalence of thyroid disorders after introduction of mandatory universal salt iodization for 16 years in China: a cross-sectional study in 10 cities. *Thyroid*.

[2] Aghini Lombardi F., Fiore E., Tonacchera M. (2013). The effect of voluntary iodine prophylaxis in a small rural community: the pescopagano survey 15 years later. *The Journal of Clinical Endocrinology & Metabolism*.

[3] Leese G. P., Flynn R. V., Jung R. T., Macdonald T. M, Murphy M. J, Morris A. D (2008). Increasing prevalence and incidence of thyroid disease in tayside, scotland: the thyroid epidemiology audit and research study (TEARS). *(Clinical Endocrinology*.

[4] Medici M., Porcu E., Pistis G. (2014). Identification of genetic loci associated with thyroid peroxidase antibodies and clinical thyroid disease. *PLoS Genetics*.

[5] Chen C., Xu H., Chen Y. (2017). Iodized salt intake and its association with urinary iodine, thyroid peroxidase antibodies, and thyroglobulin antibodies among urban Chinese. *Thyroid*.

[6] Caturegli P., De Remigis A., Rose N. R. (2014). Hashimoto thyroiditis: clinical and diagnostic criteria. *Autoimmunity Reviews*.

[7] Brčić L., Barić A., Gračan S. (2016). Association of established thyroid peroxidase autoantibody (TPOAb) genetic variants with Hashimoto’s thyroiditis. *Autoimmunity*.

[8] Pedersen I. B., Knudsen N., Carlé A. (2011). A cautious iodization programme bringing iodine intake to a low recommended level is associated with an increase in the prevalence of thyroid autoantibodies in the population. *Clinical Endocrinology*.

[9] Bai X., Shi P., Liu Y. (2014). Society: realizing China’s urban dream. *Nature*.

[10] Chen H., Liu Y., Li Z. (2017). Urbanization, economic development and health: evidence from China’s labor-force dynamic survey. *International Journal for Equity in Health*.

[11] Han B., Chen Y., Cheng J. (2018). Comparison of the prevalence of metabolic disease between two types of urbanization in China. *Front. Endocrinol (Lausanne)*.

[12] Armstrong A. D. C., Ladeia A. M. T., Marques J. (2018). Urbanization is associated with increased trends in cardiovascular mortality among indigenous populations: the PAI study. *Arquivos brasileiros de cardiologia*.

[13] Wen D., Wen X., Yang Y. (2018). Urban rural disparity in female breast cancer incidence rate in China and the increasing trend in parallel with socioeconomic development and urbanization in a rural setting. *Thoracic Cancer*.

[14] Wang R., Xue D., Liu Y. (2018). The relationship between urbanization and depression in China: the mediating role of neighborhood social capital. *International Journal for Equity in Health*.

[15] Hu S., Rayman M. P. (2017). Multiple nutritional factors and the risk of Hashimoto’s thyroiditis. *Thyroid*.

[16] Dayan C. M., Daniels G. H. (1996). Chronic autoimmune thyroiditis. *New England Journal of Medicine*.

[17] Weetman A. P. (2004). Autoimmune thyroid disease. *Autoimmunity*.

[18] Hasham A., Tomer Y. (2012). Genetic and epigenetic mechanisms in thyroid autoimmunity. *Immunologic Research*.

[19] Schultheiss U. T., Teumer A., Medici M. (2015). A genetic risk score for thyroid peroxidase antibodies associates with clinical thyroid disease in community-based populations. *The Journal of Clinical Endocrinology & Metabolism*.

[20] Duntas L. H. (2011). Environmental factors and thyroid autoimmunity. *Annales d’Endocrinologie*.

[21] Hewagama A., Richardson B. (2009). The genetics and epigenetics of autoimmune diseases. *Journal of Autoimmunity*.

[22] Burek C. L., Talor M. V. (2009). Environmental triggers of autoimmune thyroiditis. *Journal of Autoimmunity*.

[23] China National Bureau of Statistics, Statistical Communiqué of China on 2015 National Economic and Social Development, 2016, http://www.stats.gov.cn/tjsj/zxfb/201602/t20160229_ 1323991. html

[24] de Freitas C. U., Grimaldi Campos R. A., Rodrigues Silva M. A. F. (2010). Can living in the surroundings of a petrochemical complex be a risk factor for autoimmune thyroid disease?. *Environmental Research*.

[25] Camargo R. Y. A., Tomimori E. K., Neves S. C., Knobel M., Medeiros-Neto G. (2006). Prevalence of chronic autoimmune thyroiditis in the urban area neighboring a petrochemical complex and a control area in Sao Paulo, Brazil. *Clinics*.

[26] Langer P., Kočan A., Tajtáková M. (2008). Increased thyroid volume, prevalence of thyroid antibodies and impaired fasting glucose in young adults from organochlorine cocktail polluted area: outcome of transgenerational transmission?. *Chemosphere*.

[27] Mizokami T., Wu Li A., El-Kaissi S., Wall J. R. (2004). Stress and thyroid autoimmunity. *Thyroid*.

[28] Pedersen I. B., Laurberg P., Knudsen N. (2008). Smoking is negatively associated with the presence of thyroglobulin autoantibody and to a lesser degree with thyroid peroxidase autoantibody in serum: a population study. *European Journal of Endocrinology*.

[29] Effraimidis G., Tijssen J. G. P., Wiersinga W. M. (2009). Discontinuation of smoking increases the risk for developing thyroid peroxidase antibodies and/or thyroglobulin antibodies: a prospective study. *The Journal of Clinical Endocrinology & Metabolism*.

