# Supplementary material for: Influence of Rapid Urbanization on Thyroid Autoimmune Disease in China
Source: Int J Endocrinol. 2021 Jun 2;2021:9967712. doi: 10.1155/2021/9967712 (PMC8189768; doi:10.1155/2021/9967712)
Supplement: Supplementary Materials — Supplementary Table 1: description of four SNPs. Supplementary Table 2: comparison of TPOAb and TGAb positivity in two locations. [file 9967712.f1.docx]

Supplemental Table 1. Description of four SNPs.

|  | variant | chr | gene | major allele/minor allele | Effect allele frequency | *P* for HWE |
| --- | --- | --- | --- | --- | --- | --- |
| SNP 1 | rs9277555 | 6 | HLA-DPB1 | A/G | 0.49 | 0.909 |
| SNP 2 | rs11675434 | 2 | TPO | C/T | 0.31 | 0.957 |
| SNP 3 | rs301799 | 1 | RERE | T/C | 0.17 | 0.932 |
| SNP 4 | rs3094228 | 6 | HCP5, MICA | A/G | 0.15 | 0.959 |

Supplemental Table 2. Comparison of TPOAb and TGAb positivity in two locations.

|  | TPOAb positive | | | TGAb positive | | |
| --- | --- | --- | --- | --- | --- | --- |
|  | SY | NJ | P | SY | NJ | P |
| overall | 120/1546 | 177/1399 | <0.001 | 135/1546 | 228/1399 | <0.001 |
| Age |  |  |  |  |  |  |
| <40 | 19/236 | 18/162 | 0.302 | 18/236 | 30/161 | 0.001 |
| 40-50 | 32/342 | 34/257 | 0.134 | 37/342 | 46/257 | 0.013 |
| 50-60 | 23/391 | 44/373 | 0.004 | 30/391 | 49/373 | 0.013 |
| 60-70 | 35/410 | 56/432 | 0.039 | 36/410 | 72/433 | 0.001 |
| ≥70 | 11/167 | 25/175 | 0.020 | 14/167 | 31/175 | 0.011 |
| Gender |  |  |  |  |  |  |
| Male | 30/694 | 31/445 | 0.053 | 33/694 | 20/446 | 0.832 |
| Female | 90/852 | 146/954 | 0.003 | 102/852 | 208/953 | <0.001 |
